# Changes in brain gray matter volume in nasopharyngeal carcinoma patients after radiotherapy in long-term follow-up

**DOI:** 10.1016/j.bjorl.2023.01.005

**Published:** 2023-02-08

**Authors:** Wenjia Zhu, Fu Chen, Dongming Yin, Keguang Chen, Shengzi Wang

**Affiliations:** a Fudan University, Shanghai Medical College, Huashan Hospital, Department of Radiotherapy, Shanghai, China; b Eye & ENT Hospital of Fudan University, Department of Radiation Oncology, Shanghai, China; c Zhongshan Hospital Fudan University, Department of Otorhinolaryngology-Head and Neck Surgery, Shanghai, China

**Keywords:** Nasopharyngeal carcinoma, Voxel-based morphological analysis, Gray matter volume, Radiotherapy

## Abstract

**Objectives:**

The aim of this study was to examine the changes in gray matter in nasopharyngeal carcinoma patients with normal hearing (Group 1) and nasopharyngeal carcinoma patients with hearing loss (Group 2) after radiotherapy using voxel-based morphological analysis and to analyze the relationship with the radiation doses of the temporal lobe.

**Methods:**

21 patients in Group 1, 14 patients in Group 2, and 21 healthy volunteers were selected. All participants underwent an otologic examination and three-dimensional magnetization preparatory rapid acquisition gradient echo sequence scan. The correlation between the variation of whole brain gray matter volume and the doses of the temporal lobe was analyzed by Data Processing & Analysis for Brain Imaging software.

**Results:**

Compared with the normal control group, the brain areas with reduced gray matter volume in nasopharyngeal carcinoma patients after radiotherapy were mainly in the left posterior cerebellar lobe (*T* = −8.797), left insular lobe (*T* = −7.96), and the right insular lobe (*T* = −6.632). Compared to Group 1, the brain areas of Group 2 patients with reduced gray matter volume were mainly in the left superior temporal gyrus (*T* = −2.366), left olfactory bulb (*T* = −2.52), left Rolandic operculum (*T* = −2.431), and right olfactory bulb (*T* = −3.100). Compared with Group 1, the brain areas of Group 2 patients with increased gray matter volume were mainly in the left calcarine sulcus (*T* = 3.425) and right calcarine sulcus (*T* = 3.169). There were no correlations between the changes of brain gray matter volume and the radiation doses of the temporal lobe in both Group 1 and Group 2.

**Conclusions:**

The radiotherapy may cause the changes of brain areas associated with cognitive function in nasopharyngeal carcinoma in a long-term follow-up. At the same time, nasopharyngeal carcinoma patients with the radiation-induced hearing loss had abnormal gray matter volumes in the auditory center and other sensory centers. Our findings might provide new understanding into the pathogenesis of radiation-induced brain damage in normal-appearing brain tissue. Yet this exploratory study should be taken with caution.

## Highlights

• The change in brain gray matter volume in NPC patients after radiotherapy in long-term follow-up were studied.
• NPC patients with the radiation-induced hearing loss had abnormal brain gray matter volume in the auditory center and other sensory centers.
• Our findings might provide new understanding into the pathogenesis of radiation-induced brain damage in normal-appearing brain tissue.

## Introduction

In addition to ear pain, tinnitus, secretory otitis media, deafness, and other ear complications, radiation-induced encephalopathy is one of the most serious complications after radiotherapy for Nasopharyngeal Carcinoma (NPC)^1^, ^2^. At present, the pathogenesis of radiation-induced encephalopathy in NPC is still unclear, and its diagnosis mainly depends on Computed Tomography (CT) or Magnetic Resonance Imaging (MRI). However, when patients suffer from the radiation-induced encephalopathy, it is often irreversible and greatly affects their normal life. Therefore, attention has been focused on how to prevent its early occurrence. Functional Magnetic Resonance Imaging (fMRI) is a new neuroimaging method, which plays important role in brain function study because of its advantages of being non-invasive, high resolution, intuitive, repeatable research for individual subjects and patients.

Voxel-Based Morphometry (VBM) provides an automated quantitative analysis of the distribution of Gray Matter (GM) and may be applied to individuals and to groups of patients^3^. This method has been widely used in the research of Alzheimer’s disease and other diseases^4^, ^5^. The previous studies^6^ using fMRI have found abnormalities in the brain microstructure, perfusion, and metabolism of patients with NPC who did not show abnormalities in conventional MRI after radiotherapy. However, there are few reports on the effects of radiotherapy on the normal-appearing GM structure in patients with NPC and on the changes of GM in patients with sensorineural hearing loss after radiotherapy.

Thus, the aim of this study was to examine the changes in GM in NPC patients with normal hearing and NPC patients with hearing loss after radiotherapy using Voxel-Based Morphological analysis (VBM) and to analyze the relationship with the minimum, maximum, and mean doses of the temporal lobe.

## Methods

### Subjects

A retrospective analysis of 35 patients with NPC treated at a large public university from 2006 to 2015 was performed. Patients were selected based on the following selection criteria: 1) Histologically diagnosed as having NPC stages I–IVb according to the American Joint of Cancer Committee (2010), 2) Radiation therapy as the main treatment, 3) Hearing test report before treatment shows normal hearing, followed by complete hearing test after radiotherapy, 4) FMRI examinations after follow-up, and 5) Age ≤60 years old when they were diagnosed with NPC to minimize age-related sensorineural deafness. Exclusion criteria included the following: 1) History of hypertension or diabetes, 2) Local recurrence or distant metastasis at follow-up, 3) Diseases of the middle and inner ear, and 4) Past history of radiation treatment in head and neck. There are 21 healthy volunteers who were no obvious abnormalities found in a conventional head MRI and served as a control group.

### Radiation therapy

**Figure 1:**
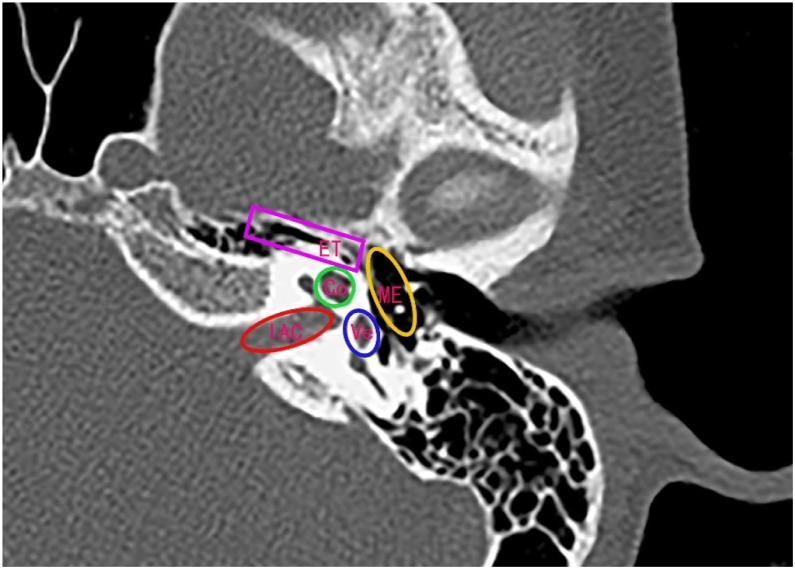
Delineation of the Eustachian Tube (ET), Middle Ear (ME), Vestibular (Vs), Cochlear (Co), and Internal Auditory Canal (IAC).

All selected NPC patients received curative Intensity Modulated Radiotherapy (IMRT). Briefly, patients were positioned supine and immobilized from head to neck with a thermoplastic mask in both computed tomography simulation and treatment delivery. The use of the GE HiSpeed type CT simulator (GE, Fairfield, CT) proceeded CT scanning. Scans were performed on a three-dimensional CT simulator with a 2.5 mm slice thickness for the primary site, a 2.5 mm slice thickness for auditory organ surface, a 1 mm slice thickness for reconstruction, and a 5 mm slice thickness for the neck. Scanning ranged from head to 2 cm below sternal notch. The data was transferred to a planning system (ADAC pinnacle 3, version 7.0, Milpitas, CA). The tumor target area and normal organs were delineated by the clinician according to the International Commission Radiological Units and Measurements Report 50. Organs at risk such as Eustachian Tube (ET), ME, Vestibular (Vs), Cochlea (Co), and Internal Auditory Canal (IAC) were delineated from the planning computed tomography (Fig. 1). We planned to develop primary tumor and upper neck with 9-field IMRT. The total doses were 67.5–69.75 Gy (2.2–2.25 Gy per time, 5 times per week) in Gross Tumor Volume (GTV), 58–60 Gy (2 Gy per time, 5 times per week) in Clinical Target Volume 1 (CTV1), and 54 Gy (1.8 Gy per time, 5 times per week) in CTV2 (upper neck CTV). For patients with N1 and N2 stages, the lower neck was irradiated with a half-field tangential line to 50–54 Gy (fractional dose 1.80–2 Gy). For N0 stage patients, the lower neck was not irradiated.

### Hearing assessment

Pure tone air and Bone Conduction (BC) audiometry, otoscopy, and tympanometry were performed. The air conduction threshold was measured at 0.125–8 kHz, and the BC threshold was measured at 0.25–4 kHz. Sensorineural hearing loss was defined as the hearing threshold was >25 dB at low-frequency from 0.5 kHz to 2 kHz and/or at high-frequency 4 kHz in one or both ears.

### Functional magnetic resonance imaging

All MRI scans were performed on a 3.0 T MR scanner (Siemens Magnetom Tim Trio, Erlangen, Germany) equipped in the Department of Radiology, EENT hospital of Fudan University. The routine imaging studies, including axial T1- and T2-weighted images and T2-FLAIR images, were obtained for every subject to detect any clinically silent lesions. A high-resolution three-dimensional Magnetization Prepared Rapid Acquisition Gradient Echo (MPRAGE) T1-weighted sequence was used to acquire MR images over the whole brain. MR imaging parameters applied in this study were repetition time of 2530 ms, echo time of 2.34 ms, field of view of 256 × 256 mm^2^, flip angle of 7°, with interslice gap of 1 mm. All subjects with abnormal signal intensity or overt brain atrophy in whole-brain GM were excluded.

### Voxel-based morphological (VBM) analysis data processing

VBM data were processed and analyzed using the SPM12 and CAT12 software. T1-weighted images were corrected for bias-field inhomogeneities, spatially normalized to the Montreal Neurological Institute standard template space, and segmented into GM, WM, and cerebrospinal fluid, within a unified model including high-dimensional DARTEL normalization. Segments were further refined using adaptive maximum a posteriori estimation, which account for partial volume effects and by applying a hidden Markov random field model. The voxel resolution after normalization was 1.5 × 1.5 × 1.5 mm^3^. Finally, the unmodulated normalized GM maps were smoothed with a standard 8 mm full-width-at-half-maximum isotropic Gaussian kernel and used for further statistical analyses of differences in GM volume between both groups.

### Statistical analysis

Age, gender and total intracranial volume served as covariates of no interest to remove the possible effect of these parameters on GM volume. A two-sample *t*-test was used to detect the differences of smooth GM images between the patients with NPC and the healthy controls using SPM12 software. AlphaSim based on Monte Carlo simulation was applied for multiple comparison correction. After correction, A p-value less than 0.05 (two-sided) was deemed significant. Data Processing & Analysis for Brain Imaging software was used to analyze the correlation between temporal lobe exposure dose and the brain GM volume. A p-value less than 0.05 (two-sided) was considered statistically significant.

## Results

In this study, A total of 35 NPC patients were included who had normal hearing before radiotherapy, had complete hearing data before and after radiotherapy, and underwent FMRI examinations after follow-up. Among them, 21 NPC patients who had the normal hearing after radiotherapy were defined as Group 1, including 13 males and 8 females. The patients were between 21 and 55 years of age. The median age was 42 years old, including T1 23.81% (5/21), T2 47.62% (10/21), T3 23.81% (5/21), T4 4.76% (1/21), 4.76% (1/21) stage I, 28.57% (6/21) stage II, 61.90% (13/21) stage III, and 4.76% (1/21) stage IV. The follow-up time was 38–131 months (median follow-up time 71 months).

The other 14 NPC patients who had the sensorineural hearing loss after radiotherapy were defined as Group 2, including 13 males and 1 female. The patients were between 30 and 52 years of age. The median age was 41 years old, including T1 21.43% (3/14), T2 42.86% (6/14), T3 14.29% (2/14), T4 21.43% (3/14), 7.14% (1/14) stage I, 42.86% (6/14) stage II, 21.43% (3/14) stage III, and 28.57% (4/14) stage IV. The follow-up time ranged from 22 to 157 months (median follow-up time 84 months) (Table 1). There were 21 healthy volunteers, including 10 males and 11 females, ages 26–58 years (median age 38.35 years).

**Table 1:** Clinical characteristics of nasopharyngeal carcinoma patients after radiotherapy.

Variable	Group 1, *n* (%)	Group 2, *n* (%)
Median age, yr. (range)	42 (21–55)	41 (30–52)
Sex		
Male	13 (61.90%)	13 (92.86%)
Female	8 (38.10%)	1 (7.14%)
T staging		
T1	5 (23.81%)	3 (21.43%)
T2	10 (47.62%)	6 (42.86%)
T3	5 (23.81%)	2 (14.29%)
T4	1 (4.76%)	3 (21.43%)
Clinical stages		
Stage I	1 (4.76%)	1 (7.14%)
Stage II	6 (28.57%)	6 (42.86%)
Stage III	13 (61.90%)	3 (21.43%)
Stage IV	1 (4.76%)	4 (28.57%)
Follow-up time, mth. (range)	71(38–131)	84(22–157)

Group 1, The normal hearing after radiotherapy; Group 2, The sensorineural hearing loss after radiotherapy.

**Figure 2:**
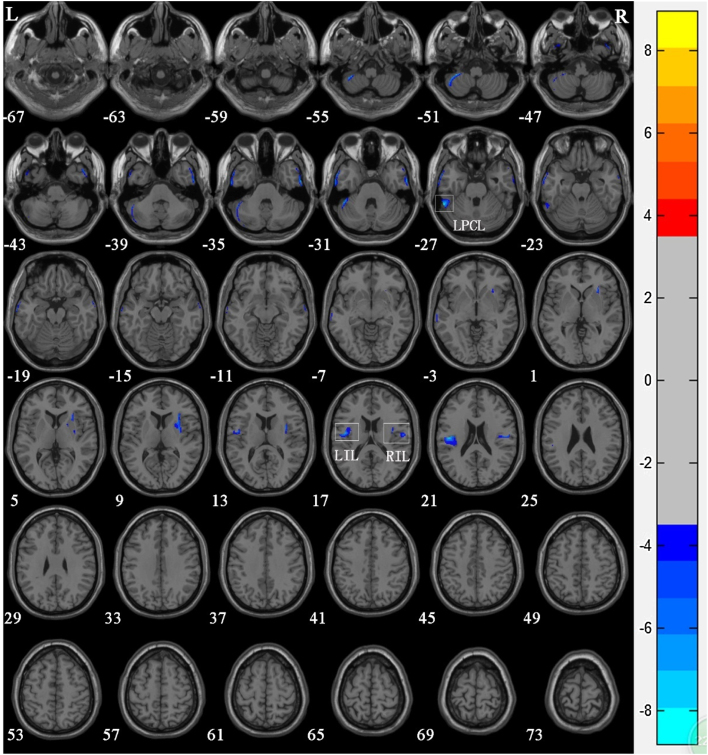
Voxel-based morphometry shows a reduction in gray matter volume in NPC patients after radiotherapy compared to that of the normal control Group. The numbers in the figure are the *z*-axis coordinates of Montreal Neurological Institute space. The color bar is *t*-value. The images show areas of significantly reduced gray matter volume in Left Posterior Cerbellar Lobe (LPCL), Right Insular Lobe (RIL), and Left Insular Lobe (LIL).

There was no significant difference in age among members of the control group, Group 1, and Group 2. VBM results showed that compared with the control group, significant reductions in GM volume in NPC patients after radiotherapy were mainly in the left posterior cerebellar lobe (*T* = −8.797), left insular lobe (*T* = −7.96), and the right insular lobe (*T* = −6.632) (Table 2 and Fig. 2).

**Table 2:** Regions of reduced gray matter volume in nasopharyngeal carcinoma patient group in comparison with the control group.

Brain region	MNI coordinates			Voxel size	Peak *t-*value
	X	Y	Z		
Left posterior cerbellar lobe	−54	−45	−28	356	−8.797^a^
Right insular lobe	52	−12	19	153	−6.632^a^
Left insular lobe	−43	−19	22	225	−7.960^a^

MNI, Montreal Neurological Institute.

a *p* <  0.05 (after Alphasim correction).

**Figure 3:**
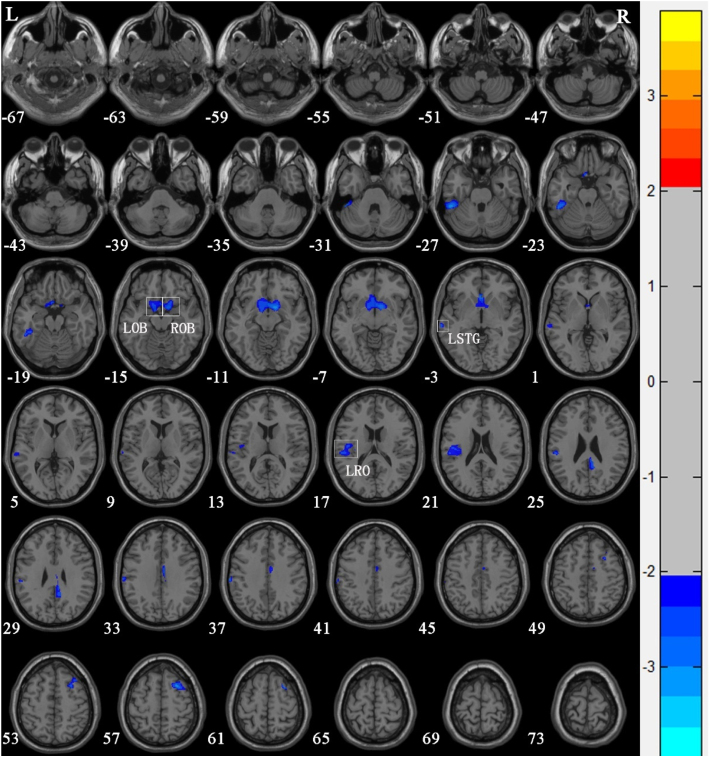
Voxel-based morphometry shows a reduction in gray matter volume in nasopharyngeal carcinoma patients with radiation-induced hearing loss compared to nasopharyngeal carcinoma patients with normal hearing after radiotherapy. The numbers in the figure are the *z*-axis coordinates of Montreal Neurological Institute space. The color bar is *t*-value. The images show areas of significantly reduced gray matter volume in Left Olfactory Bulb (LOB), Right Olfactory Bulb (ROB), left Superior Temporal Gyrus (LSTG), and Left Rolandic Operculum (LRO).

**Figure 4:**
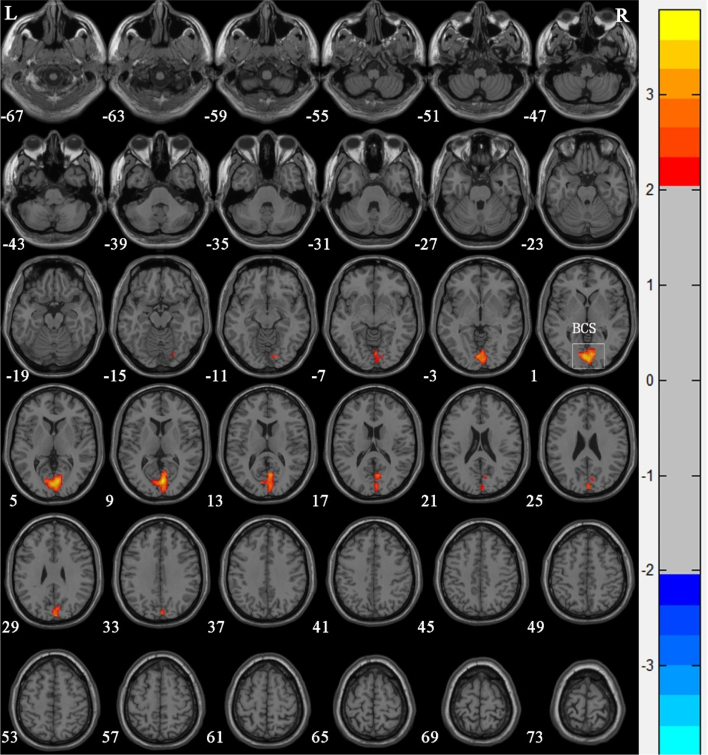
Voxel-based morphometry shows an increase in gray matter volume in nasopharyngeal carcinoma patients with radiation-induced hearing loss compared to nasopharyngeal carcinoma patients with postradiotherapy normal hearing after radiotherapy. The numbers in the figure are the *z*-axis coordinates of Montreal Neurological Institute space. The color bar is *t*-value. The images show areas of significantly increased gray matter volume in both left and right Calcarine Sulcus (BCS).

Compared with Group 1, the brain areas with significantly reduced GM volume in Group 2 patients were mainly in the left superior temporal gyrus (*T* = −2.366), the left olfactory bulb (*T* = −2.52), the left Rolandic operculum (*T* = −2.431), and the right olfactory bulb (*T* = −3.10) (Table 3 and Fig. 3). Compared with Group 1, the brain areas with significantly increased GM volume in Group 2 patients were mainly in the left calcarine sulcus (*T* = 3.425) and the right calcarine sulcus (*T* = 3.169) (Table 3 and Fig. 4). There were no correlations between the changes of brain GM volume and the radiation doses of the temporal lobe in both Group 1 and Group 2.

**Table 3:** Regions of altered gray matter volume in nasopharyngeal carcinoma patients with radiation-induced hearing loss compared to nasopharyngeal carcinoma patients with normal hearing after radiotherapy.

Brain region	MNI coordinates			Voxel size	Peak *t-*value
	X	Y	Z		
Left superior temporal gyrus	−65	−27	7	144	−2.366^a^
Left olfactory bulb	−13	7	−12	420	−2.520^a^
Left rolandic operculum	−48	−16	19	460	−2.431^a^
Right olfactory bulb	12	7	−10	258	−3.100^a^
Left calcarine sulcus	−5	−83	−1	1127	3.425^a^
Right calcarine sulcus	6	−86	−1	1072	3.169^a^

MNI, Montreal Neurological Institute.

a *p* <  0.05 (after Alphasim correction).

## Discussion

The incidence of radiation encephalopathy after radiotherapy for NPC is lower than that in conventional radiotherapy because of the use of IMRT technology^7^, ^8^. However, functional MRI studies on NPC after IMRT have showed that the brain volume for patients without radiation encephalopathy have decreased compared to those of healthy persons, suggesting that although IMRT reduces the radiation dose in normal brain tissue, the effect of radiotherapy on brain tissue still exists^9^, ^10^.

Previous study found that some brain regions will change dynamically with the passage of time after radiotherapy^6^, ^11^. It is essential for long-term research on the brain function of patients. The median follow-up time of the target population in our study was more than 5 years. In our study, compared with the control group, the reduced GM volume in NPC patients after radiotherapy were mainly in the left posterior cerebellar lobe and the bilateral insular lobe. Traditionally, the cerebrum has been thought of as the brain’s main area for cognitive function, while the cerebellum is responsible for regulating and coordinating movement, posture, and balance. However, recent studies have shown that the cerebellum may also play a role in neurocognitive function^12^, ^13^, ^14^. In the study of glioma radiotherapy, it was found that there was a significant correlation between radiation dose of the posterior cerebellum lobe and neurocognitive impairment. Gan et al.^15^ studied 10 cases of patients with head and neck squamous cell carcinoma who were receiving radiotherapy and found that the average score of all patients in the cognitive field was significantly lower than expected. The insular lobe is not only the primary taste cortex^16^, but also the major cortex of the interoceptive signal, which is crucial for emotional perception. In this study, it was found that compared with the control group, the GM volume in the posterior cerebellar lobe and insular lobe was reduced in NPC patients, which also indicated that the patients might have had a decline in cognitive and emotional regulation after radiotherapy^17^, ^18^.

Comparing with Group 1, we found that the brain regions with reduced GM volume in Group 2 patients were mainly in the left superior temporal gyrus, bilateral olfactory bulb, and left Rolandic operculum. We speculated that sensorineural deafness in NPC patients after radiotherapy might lead to decreased function of the primary auditory cortex. Meanwhile, the included population was right-handed, and the left cerebral hemisphere was mainly manifested in language function, so it was manifested as reduced GM volume of left superior temporal gyrus. At the same time, we also found that the GM volume of bilateral olfactory bulbs decreased. In some studies, scholars have found auditory sensory convergence in olfactory nodules, which would cause the decrease of olfactory bulb volume in patients with impaired auditory cortex function and reduced auditory signals. Our results were consistent with those from the study of Veyseller et al.^19^, who compared the olfactory area of 24 NPC patients after radiotherapy with those of a control group and found that the olfactory bulb volume of patients with NPC after radiotherapy was smaller than that of the control group. Blefari et al.^20^ proposed that the Rolandic operculum processes integrated exteroceptive-interoceptive signals that are necessary for interoceptive awareness as well as bodily self-consciousness. Xu et al.^21^ conducted an fMRI study on patients with sensorineural hearing loss and found that the functional connectivity of the Rolandic operculum was reduced in patients, suggesting that the Rolandic operculum may also be involved in sensory integration and emotional and cognitive impairments caused by sensorineural hearing loss.

At the same time, we found that compared with Group 1, the GM volume of Group 2 patients was increased in the bilaterally calcarine sulcus. The superior and inferior cortexes of the calcarine sulcus are considered as the visual center and the visual union cortex, which receives visual stimuli directly and processes basic visual information. The increase of GM volume in those regions suggests that the visual cortex is functionally compensated in patients with sensorineural hearing loss and auditory cortex volume reduction. Regarding Group 1 and Group 2, there were not any significant correlations between GM volume changes and temporal lobe exposure doses. As reported in the literature^22^, the volume and location of radionecrosis had an influential impact on the pattern of cognitive impairment found in patients with NPC. There are also studies^23^ that show radiotherapy for nasopharyngeal carcinoma seemed to have adverse but insignificant effects on the cognitive functions of the patients. In our study, patients also showed no obvious cognitive impairment during the follow-up. Even so, the shortcoming of this study is that there is lack of attention and study on the relationship between the change in brain gray matter volume and cognitive function. Yet this is an exploratory study, whose findings should therefore be taken with caution and further study.

## Conclusion

The radiotherapy may cause the changes of brain areas associated with cognitive function in NPC patients in a long-term follow-up. At the same time, NPC patients with the radiation-induced hearing loss had abnormal GM volumes in the auditory center and other sensory centers. Our findings might provide new understanding into the pathogenesis of radiation-induced brain damage in normal-appearing brain tissue.

## Ethics statement

The study was approved by the Clinical Research Ethics Committee of the Eye & ENT Hospital of Fudan University. All procedures performed in studies involving human participants were in accordance with the ethical standards of the institutional and/or national research committee and with the World Medical Association Declaration of Helsinki (version 2002) and the additional requirements. Informed consent was obtained from all individual participants included in the study.

## Conflicts of interest

The authors declare no conflicts of interest.
